# Machine Learning Identifies Smartwatch-Based Physiological Biomarker for Predicting Disruptive Behavior in Children: A Feasibility Study

**DOI:** 10.1089/cap.2023.0038

**Published:** 2023-11-15

**Authors:** Magdalena Romanowicz, Kyle S. Croarkin, Rana Elmaghraby, Michelle Skime, Paul E. Croarkin, Jennifer L. Vande Voort, Julia Shekunov, Arjun P. Athreya

**Affiliations:** ^1^ Department of Psychiatry and Psychology, Mayo Clinic, Rochester, Minnesota, USA.; ^2^ Mayo Clinic Children's Research Center, Rochester, Minnesota, USA.; ^3^ Department of Molecular Pharmacology and Experimental Therapeutics, Mayo Clinic, Rochester, Minnesota, USA.; ^4^ Department of Psychiatry and Behavioral Sciences, University of Washington, Seattle, Washington, USA.

**Keywords:** wearable, smartwatch, prediction, child behavior

## Abstract

**Objective:**

Parents frequently purchase and inquire about smartwatch devices to monitor child behaviors and functioning. This pilot study examined the feasibility and accuracy of using smartwatch monitoring for the prediction of disruptive behaviors.

**Methods:**

The study enrolled children (*N* = 10) aged 7–10 years hospitalized for the treatment of disruptive behaviors. The study team completed continuous behavioral phenotyping during study participation. The machine learning protocol examined severe behavioral outbursts (operationalized as episodes that preceded physical restraint) for preparing the training data. Supervised machine learning methods were trained with cross-validation to predict three behavior states—calm, playful, and disruptive.

**Results:**

The participants had a 90% adherence rate for per protocol smartwatch use. Decision trees derived conditional dependencies of heart rate, sleep, and motor activity to predict behavior. A cross-validation demonstrated 80.89% accuracy of predicting the child's behavior state using these conditional dependencies.

**Conclusion:**

This study demonstrated the feasibility of 7-day continuous smartwatch monitoring for children with severe disruptive behaviors. A machine learning approach characterized predictive biomarkers of impending disruptive behaviors. Future validation studies will examine smartwatch physiological biomarkers to enhance behavioral interventions, increase parental engagement in treatment, and demonstrate target engagement in clinical trials of pharmacological agents for young children.

## Introduction

Evidence-based interventions such as parent–child interaction therapy (PCIT) reduce behavioral and emotional symptoms in children by improving parent–child relationships through the implementation of specific rules taught over a multiweek period (Chaffin et al., 2011; Eyberg et al., 2001; Hood and Eyberg, 2003).

Despite the widespread availability of PCIT, there are significant challenges: (1) the effectiveness of PCIT is contingent upon parents making a concerted effort to remember the rules imparted by a provider in engaging with their children and (2) families from rural areas and under-represented populations are less likely to have access to and utilize evidence-based therapies when it is available (Bauermeister et al., 2003; Bell et al., 2021; McIver et al., 2021; Rubinstein et al., 1985; Shi et al., 2021). Therefore, there is a need for innovations that utilize technologies to enhance PCIT's accessibility and effectiveness in differing evolving social contexts.

In the era of digital health (the use of communication and information technology for passive or remote monitoring to deliver precision health care), there is an opportunity to improve access and effectiveness of behavioral interventions in children and adolescents, including PCIT. Smartwatches measure individual functioning in real time and provide opportunities to identify point-of-care smartwatch biomarkers in young children that can provide prompts for parents of child's impending behavioral outbursts. Accessible digital tools may have the promise of increasing parental engagement for improved outcomes.

The growth in children's smartwatch market (valued at $1.15 billion in 2020) is largely driven by parental interest to track their child's functioning (Leroux et al., 2021). Recent research has demonstrated that wearables (e.g., Google glass) can be used to promote positive behavior in children diagnosed with autism spectrum disorder (Al Mamun et al., 2016; Carpenter et al., 2021; O'Brien et al., 2020; Pollreisz and TaheriNejad, 2017; Stephenson and Limbrick, 2015; Voss et al., 2019).

Other independent studies have demonstrated parental acceptance (Mackintosh et al., 2019), tolerance (Mackintosh et al., 2019), clinical validations of smartwatch activity in children aged 4–7 years (Byun et al., 2018), and other physiological measurements (Chinoy et al., 2021; Evenson and Spade, 2020; Stevens and Siengsukon, 2019; Tedesco et al., 2019).

**FIG. 1.**
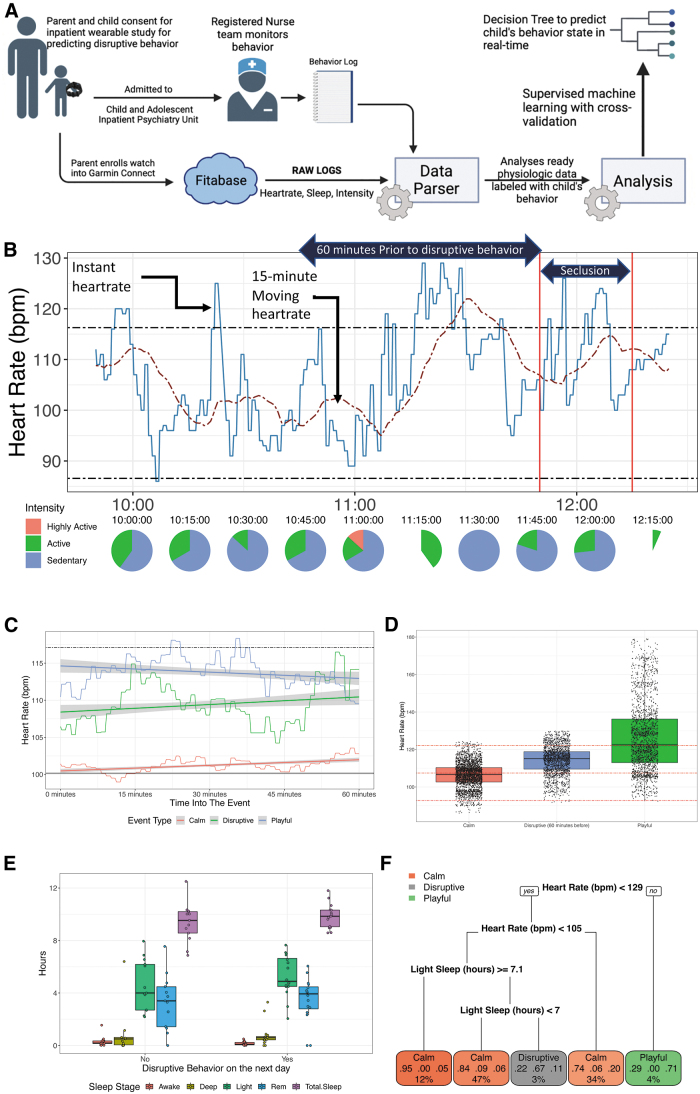
**(A)** Study design and analytical workflow. **(B)** Heart rate (solid blue line—instant heart rate; dashed red line—15-minute moving average) and intensity variations during the onset of a disruptive behavior. **(C)** Variations of moving average heart rate (and associated regression with 95% confidence interval) across behavior phenotypes in a 60-minute window. Solid black line is the average resting heart rate across all patients, and dashed black line is the average heart rate 1 standard deviation above the resting heart rate. **(D)** Instant heart rates across behavior phenotypes. The solid blue line is the instantaneous heart rate recorded each minute by the smartwatch and the horizontal dashed red line is the 15-minute moving average. The bottom and top black dashed lines are the resting heart rate (reported by smartwatch) and average heart rate 1 standard deviation above the resting heart rate. The solid vertical red lines are the start and end of the period child was in seclusion after an uncontrolled behavior outburst. **(E)** Variations in sleep on days with and without disruptive behaviors. **(F)** Decision tree to predict calm, playful, and onset of disruptive behavior. In the tree, within the predicted behavior (colored boxes as leaves of the tree), second row of numbers is proportion of events predicted by that conditional path of the tree, and the first row of numbers is the proportion of behavior events of each class predicted by the tree for the given conditional statement (e.g., in the green Playful box, 29% events are Calm; 0% events are Disruptive; 71% of events are Playful).

This pilot study (see Fig. 1A) sought to establish whether physiological data from smartwatches could predict disruptive behaviors in a sample of hospitalized children. Specifically, this pilot study focused on examining the feasibility of smartwatch monitoring in children and whether machine learning approaches with physiological smartwatch data could predict impending disruptive behaviors. It was hypothesized that a predictive model with heart rate and sleep data could predict disruptive behaviors before their manifestation.

## Methods

### Sample

Hospitalized children with disruptive behaviors (*N* = 10) aged 7–10 years were enrolled in this IRB-approved study conducted during May 2020 through December 2020. Registered nurses (RNs) of the Mayo Clinic Child and Adolescent Psychiatry inpatient unit continuously assessed and annotated behavior for 24 hours daily during hospitalization (see behavior log in Supplementary Table S1). The annotations serve as the labels for behavior to be predicted using machine learning approaches that utilize data from smartwatches. Additional study inclusion–exclusion criteria are described in Supplementary Data.

### Smartwatch enrollment

Participants wore Garmin vivosmart4 smartwatches during the inpatient hospitalization, except for times when they showered, had meals, or refused to wear the watch. The smartwatch collected motor activity (referred to as intensity by Garmin, which is minutes spent being sedentary, active, or highly active), heart rate (beats per minute), and sleep (including duration and sequence of sleep stages) data through the Garmin Connect mobile application. The smartwatch minute-level data were aggregated and collected through Fitabase. Each participant and family had an abbreviated PCIT training intervention during hospitalization.

### Behavior phenotyping

Inpatient RNs annotated behavior states as either calm, playful, or disruptive using the behavior code described in Supplementary Table S1. *Calm* (behavior codes 1–4, 7, 8) indicated that the child was sedentary while performing routine activities such as reading, lying down, watching movie, talking/interacting with others, or doing their daily homework. *Playful* (behavior codes 5, 6) indicated that the child was engaged in a nondisruptive activity of elevated heart rate such as exercising (e.g., on a treadmill), playing a sport (e.g., basketball), or playing with other children (e.g., outdoor gymnasium).

*Disruptive* (behavior codes 9–11) indicated that the child showed uncontrolled aggression/behavior (e.g., tantrum) warranting intensive behavioral interventions, time-outs, or restraint for safety. For each of the calm, playful, and disruptive phenotypic annotations on a given day, the corresponding average heart rate in the preceding 60 minutes and the durations of sleep stages from the previous night were captured with the smartwatches (see Supplementary Table S2).

### Machine learning for predicting disruptive behavior

Decision trees were trained to use heart rate (60-minute average) and sleep data to predict calm, playful, or impending disruptive behaviors (that warranted physical restraint; see Table 1 for distribution of disruptive behavior across subjects). Decision trees are nonparametric supervised machine learning algorithms that generate conditional dependency statements (that can be programmed into low-cost smartphones connecting to a smartwatch) to indicate the likelihood of an impending event. Tenfold cross-validation with chi-square pruning (using RPART package in R) was conducted to derive the optimal split points for each level of the tree to reach the final decision of whether the physiological data were predictive of either of the three behavior states.

**Table 1.** Cohort Summary

Subject ID	Gender	Age	Marital status of parents	Days in the study	Hours watch worn during day	Watch wear time (% per day)	Number of disruptive events	Diagnosis	Medications at discharge
1	F	9	Separated	15	151.5	87	12^a^	**ODD**^b^, ADHD	Amphetamine/dextroamphetamine salts, clonidine
2	M	10	Separated	6	123.5	95	4	**ODD**^b^, ADHD, Developmental Speech Language Disorder	Aripiprazole, atomoxetine, clonidine, divalproex sodium, fluoxetine
3	M	8	Married	10	166.6	87	1	**ODD**^b^, ADHD	Methylphenidate, sertraline, melatonin
4	F	9	Unknown	6	102.4	83	2	**ODD**^b^, RAD, ADHD	Guanfacine, methylphenidate, quetiapine, melatonin, iron, carbonyl-vitamin C
5	F	8	Separated	8	139.6	93	4	**ODD**^b^, ADHD, OCD	Amphetamine/dextroamphetamine salts, escitalopram
6	M	7	Separated	4	55.9	90	0	**ODD**^b^, ADHD	Clonidine, quetiapine, cholecalciferol
7	M	7	Single	5	83.5	86	0	**Problem behavioral child**^b^, Anxiety disorder unspecified	Fluoxetine, multivitamin
8	M	9	Married	17	356.2	91	17^a^	**Conduct disorder**^b^, ADHD, PTSD	Lurasidone, methylphenidate
9	F	9	Single	7	87.6	96	3	**Adjustment Disorder Mixed Emotion and Conduct**^b^, Anxiety	Melatonin
10	F	7	Single	5	102.6	97	0	**Disruptive Behavior Disorder**^b^, PTSD, Developmental Speech Language Disorder	Clonidine, risperidone

^a^ Disruptive behavior events in subjects who warranted physical restraint and used in training the decision tree.

^b^ Primary diagnosis is in bold. ODD, oppositional defiant disorder; ADHD, attention deficit/hyperactivity disorder; RAD, reactive attachment disorder; OCD, obsessive compulsive disorder; PTSD, posttraumatic stress disorder.

## Results

### Smartwatch tolerability and compliance

The smartwatches were worn for an average of 7 days and 90% of the time, resulting in 1369.2 hours of physiological data (see Table 1). The study captured 29 disruptive events that resulted in physical restraint of the child for safety that were used as labels to infer smartwatch biomarkers using supervised machine learning methods. There were 267 and 47 hours where the child was either calm or playful, respectively, with no incidents of disruptive behavior for those time periods.

### Smartwatch biomarkers

#### Motor activity (intensity) variations in 60 minutes of behavior phenotypes

The Garmin vivosmart4 smartwatch provides three levels of intensity of motor activity (Inc; Garmin Data Dictionary 2020). Sedentary: little to no movement; active: engaging in a walk; and highly active: engaging in running or jumping jacks. In the 60-minute duration of when subjects were calm or playful (without disruptive behavior) or leading up to disruptive behavior, the proportion of motor activity intensities was significantly different (*p* < 2E-16, chi-square test) across the three behavior phenotypes. Figure 1B illustrates a calm period that preceded an episode of dysregulation.

#### Heart rate variations across behavior phenotypes

The moving heart rate over 60 minutes and related behavioral phenotypes are illustrated in Figure 1C (the 95% confidence interval around the mean as derived using linear regression). The heart rate during the entire 60 minutes was significantly different across the three behavior phenotypes (*p* < 2E-16). Notably, when participants were characterized as calm, there was no difference (*p* > 0.8) between the mean heart rate during the 60-minute calm period and the overall resting heart rate (the solid black line shown in Fig. 1D).

#### Sleep variations across behavior phenotypes

The Garmin vivosmart4 watch provides the duration and sequence of sleep stages (light, deep, rapid eye movement [REM], and awake) (Chinoy et al., 2021). The total sleep durations (in hours) or amount of time awake was not significantly different among participants who exhibited disruptive behavior and those who did not (*p* > 0.16). However, the durations of REM, light, and deep sleep stages were different between participants who exhibited disruptive behavior versus those who did not (*p* ≤ 0.04; see Fig. 1E). In participants who exhibited disruptive behavior, the odds (odds ratio 6) of disruptive behavior were significant (*p* < 0.05) if the duration of light sleep exceeded 4 hours, and there were no significant associations with the total sleep duration or duration of awake, deep, and REM sleep.

### Predicting behavior phenotypes by combining heart rate, intensity, and sleep measures

A decision tree derived conditional dependencies with physiological data predictive of the three behavior states (Fig. 1F). If the heart rate is >129 bpm, then there was a 71% chance of the child being playful. If the heart rate was <105 and with ≥7 hours of light sleep (the night before), then there was a 67% chance of a disruptive behavior in the next 60 minutes. In all other instances, the child was likely calm. In the 10-fold cross-validation across 10 participants, the accuracy of predicting the child's behavior state using these conditional dependencies was 80.89% (with a false-positive rate of 7.2% for disruptive behavior).

## Discussion

This pilot study demonstrated the feasibility of machine learning methods using data from smartwatches to predict disruptive behavior in children. The 10 subjects in the study had a 90% adherence rate for wearing the smartwatch for the duration of their hospitalization. Decision trees utilizing heart rate, sleep, and motor activity data derived from the smartwatch predicted child's behavior state (i.e., calm, playful, or impending disruptive behavior) with an accuracy of 80.89%.

Prior research has demonstrated parental acceptance (Mackintosh et al., 2019), tolerance (Mackintosh et al., 2019), clinical validations of smartwatch measures in children aged 4–7 years (Byun et al., 2018) and Garmin's measurements (Chinoy et al., 2021; Evenson and Spade, 2020; Stevens and Siengsukon, 2019; Tedesco et al., 2019). This digital biomarker framework will inform the development of ecologically valid decentralized interventions for improved contemporary family engagement and health.

These predictive platforms have the promise of facilitating objective reliable assessments for children with emotional and behavioral symptoms as opposed to standard questionnaires, interviews with parents recreating behavioral events, and the time burdens associated with current approaches (Abernethy et al., 2022; Harvey et al., 2022). Future innovations with the present platform will also increase the accessibility of evidence-based treatment for children and families.

Smartwatches with U.S. Food and Drug Administration (FDA)-approved features (e.g., to detect atrial fibrillation) are becoming ubiquitous across all ages for wellness monitoring and improved health-related outcomes. Cardiology practice has adopted the use of smartwatch devices for monitoring and assessment of remote cardiac monitoring (Perez et al., 2019). For example, benchmarks of clinically meaningful predictive accuracies for atrial fibrillation are established and documented for FDA applications. Child and adolescent psychiatry practice has been slow to evolve in this area despite a considerable need for more objective assessments.

Additional studies (e.g., NCT05077722 and NCT05725525) are underway to establish benchmarks and the utility of wearable devices for monitoring, decision support, augmented interventions, and predictive models. Valid and accessible digital biomarkers will have great utility for future pharmacological trials for young children.

This study has limitations. First, an inpatient monitoring study did not present specific behavioral triggers in outpatient settings (e.g., home, school, and interpersonal stressors). Biomarker thresholds and accuracies of predicted behavior states may vary between patients based on parents' perception of what constitutes disruptive behavior versus nearly homogeneous annotations of disruptive behavior marked by trained nurses. Second, only one smartwatch brand was used and additional studies with trackers from other vendors (e.g., fitbit by Google, Apple, Inc.) are needed. Third, the study used data from hours with complete measurements, hence the impact of time gaps in measurements is yet to be understood.

Fourth, it is possible that more computationally intensive algorithms may outperform prediction performance of decision trees with larger sample sizes and duration of observation. Fifth, although the study psychiatrists and medical records did not identify any medication-related side effects in the sample, the potential impact of concurrent psychotropic medications on physiological measures (including sleep disturbances) is unknown as it is highly possible that the threshold of biomarkers varies with time and improves behavior states.

Therefore, larger studies with longer duration of observation and treatment modalities are needed to further develop and refine time-series algorithms to predict impending disruptive behavior in children. Finally, digital biomarkers derived in this study need validation in an outpatient setting to guide the development of a technology-enhanced version of behavioral interventions such as PCIT.

## Clinical Significance

There is a critical societal need to better understand and leverage scalable remote technologies to foster healthy parenting practices within the framework of traditional evidence-based behavioral therapies such as PCIT. This feasibility study demonstrates the capability to introduce precision target-based (e.g., heart rate, sleep, and motor activity) technologies using smartwatch-derived biomarkers to improve effectiveness of psychosocial interventions in young children by predicting disruptive behavior in children before manifestation. Such biomarkers when successful could be utilized in delivering precision care to children and families through remote care capabilities—thereby addressing barriers to accessing specialty pediatric behavior clinics.

## Supplementary Material

Supplemental data

Supplemental data

Supplemental data
