# Supplementary material for: Machine Learning Identifies Smartwatch-Based Physiological Biomarker for Predicting Disruptive Behavior in Children: A Feasibility Study
Source: J Child Adolesc Psychopharmacol. 2023 Nov 15;33(9):387–92. doi: 10.1089/cap.2023.0038 (PMC10698791; doi:10.1089/cap.2023.0038)
Supplement: Supplemental data [file Suppl_TableS1.docx]

**Supplementary Table S1: Behavior log chart**
