# Supplementary material for: Machine Learning Identifies Smartwatch-Based Physiological Biomarker for Predicting Disruptive Behavior in Children: A Feasibility Study
Source: J Child Adolesc Psychopharmacol. 2023 Nov 15;33(9):387–92. doi: 10.1089/cap.2023.0038 (PMC10698791; doi:10.1089/cap.2023.0038)
Supplement: Supplemental data [file Suppl_Data.docx]

**Supplementary Methods**

**Inclusion and Exclusion Criteria**

Inclusion criteria were kept broad to enhance generalizability. Inclusion criteria included: psychiatrically hospitalized children aged 4-11 years old whose measure of externalizing behavior problems rated above the clinically significant range (≥120; T-score ≥ 60) (Eyberg Child Behavior Inventory- ECBI; Eyberg & Pincus, 1999). Exclusion criteria included: intellectual disability, autism spectrum disorder, psychotic disorder, foster care status, parents not consenting to the study, and the child being unable or unwilling to wear the smartwatch.
