# Supplementary material for: Machine Learning Identifies Smartwatch-Based Physiological Biomarker for Predicting Disruptive Behavior in Children: A Feasibility Study
Source: J Child Adolesc Psychopharmacol. 2023 Nov 15;33(9):387–92. doi: 10.1089/cap.2023.0038 (PMC10698791; doi:10.1089/cap.2023.0038)
Supplement: Supplemental data [file Suppl_TableS2.docx]

**Supplementary Table S2: Raw Data Extraction and Preparation**

| Smartwatch generated measure | Units | Preprocessing |
| --- | --- | --- |
| Intensity   - Sedentary - Active - Highly active | seconds | The Garmin API exposes the sequence of observed sequence of intensity of activity and duration (in seconds). For each 60-minute window, we aggregate the percent time spent in either of intensity classes. |
| Heart rate | Beats per minute (bpm) | The Garmin API exposes 4 measurements of heart rate per minute. Then each heart rate measurement is averaged with a maximum of 60 prior consecutive measurements resulting in a 15-minute moving window. The moving window provides a way to reduce the impact of instantaneous spikes in heart rate or possible measurement errors. |
| Sleep stages   - Light sleep - Deep sleep - Rapid eye movement (REM) sleep - Awake | hours | The Garmin API exposes the sequence of observed sleep stages and associated durations during the entire duration of sleep recorded on a given calendar date. For each calendar date, we aggregate the total hours of sleep, light sleep, deep sleep, REM sleep and awake. |
